# Targeting men to improve maternal and child health and nutrition: A qualitative process evaluation of a mass media campaign in Tanzania’s Lake Zone

**DOI:** 10.1371/journal.pone.0338437

**Published:** 2026-01-02

**Authors:** Dotto Kezakubi, Pieter Remes, Vianney Atugonza, Rosemary Kayanda, Abbie Clare, Ada Humphrey, Joanna Murray, Roy Head

**Affiliations:** 1 Development Media International, Mwanza, Tanzania; 2 University of Minnesota, Twin Cities, Minnesota, United States of America; 3 Development Media International, London, United Kingdom; Menzies School of Health Research: Charles Darwin University, AUSTRALIA

## Abstract

**Background:**

Undernutrition in the 1000 days following conception can cause lasting damage. In Tanzania malnutrition is directly or indirectly responsible for 35% of all deaths among children under five. The ASTUTE project, delivered in the five Lake Zone regions of Tanzania (Mwanza, Shinyanga, Geita, Kagera, Kigoma), aimed to reduce the prevalence of child stunting by improving child and maternal nutrition practices. The project incorporated a mass media campaign designed to change behaviours, with a particular emphasis on shifting gender norms and engaging men.

**Objective:**

This paper focuses on the qualitative findings relating to the impact of the campaign on men’s knowledge, attitudes, norms and behaviours.

**Methods:**

Throughout the period of radio and TV broadcasting, a qualitative process evaluation was conducted to assess the impact of the campaign on knowledge, attitudes, norms and behaviours. Between October 2017 and February 2020, a total of 59 focus group discussions were conducted with mothers, fathers and elder caregivers of a child aged under two years. Data were analyzed thematically.

**Results:**

Fathers reported that radio and TV spots increased their participation in maternal health by accompanying their partners to the ANC, supporting their nutrition intake, and reducing the workload of the mother during pregnancy. Radio and TV spots were also reported to increase men’s engagement in child health by encouraging exclusive breastfeeding and nutritious complementary feeding.

**Conclusion:**

Mass media campaigns that target men’s behaviour can have beneficial effects on maternal and child health. In particular, radio and TV messages targeting men appear to be able to mitigate some barriers arising from gender norms and women’s social position in Tanzania.

## Introduction

Adequate nutrition is fundamental to child development [1]. Undernutrition in the 1000 days following conception can cause lasting damage. It can lead to stunting, impaired cognitive development, delayed motor development, poor school performance, and reduced productivity later in life [2]. Stunting in children under 5 is an indicator of chronic, long-term undernutrition. Globally, stunting affects around 22% (149 million) of children under five, but the prevalence is markedly higher in low and middle-income countries (LMIC) [3].

Across Africa, the burden of maternal and child undernutrition remains persistent. For example, in Uganda, 33% of deaths are linked to malnutrition [4], and in Malawi, only 8% of children access the minimum acceptable diet of at least four food groups a day [5]. While in Kenya, despite the adoption of a set of high-impact Infant and Young Child Feeding (IYCF) policies and guidelines, only 39% of all children aged 6–23 months are fed in accordance with the recommendations [6]. Despite the adoption of evidence-based nutrition policies and guidelines, many African countries struggle with implementation due to entrenched gender and social norms that shape household decision-making and caregiving practices and socio-economic burden in most households [7].

In Tanzania, malnutrition, particularly undernutrition, is reflected by stunting, underweight, and wasting among children under five years [2]. In 2015, despite substantial improvement over the previous two decades, Tanzania had one of the world’s highest rates of stunting, with one in three children under the age of five classified as stunted [8]. Malnutrition is directly or indirectly responsible for 35% of all deaths among children under five in the country [2]. Effective maternal and child health practices, such as proper nutrition during pregnancy, appropriate breastfeeding, and complementary feeding, are key to preventing stunting and improving optimal child development. Tanzania has several governmental interventions, guidelines and policies in place which aim to improve maternal and child health and reduce stunting. For example, promoting nutritional behavioural change among families [9] and through strategies to achieve adequate nutritional status for all Tanzanians [10].

Mass media (radio, TV, social media) campaigns have increasingly been used as behaviour change interventions to improve health behaviours [11–14]. Studies have suggested that the ability for mass media campaigns to encourage behaviour change depends on the ability to disseminate well-defined and behaviour-focused messages to a large audience repeatedly [15–17]. The effectiveness of mass media campaigns to cost-effectively change health-related behaviours in low-income settings has previously been evidenced in two cluster randomized controlled trials (RCT) conducted in Burkina Faso [16,17].

The Addressing Stunting in Tanzania Early (ASTUTE) project aimed, over five years, to reduce stunting prevalence among children in the Lake Zone regions of Tanzania, using the Saturation+ approach that was used in effective mass media campaigns in Burkina Faso [18,19]. Four organizations worked together to design, implement, and assess ASTUTE. Interchurch Medical Assistance (IMA) World Health, the lead organization, was responsible for the technical and financial management of the project. Partnership for Nutrition in Tanzania (PANITA) worked with civil society organizations to strengthen their capacity in nutrition counselling via training, mentoring and monitoring. Development Media International (DMI) led the design and implementation of the mass media campaign via radio and television, conducted formative research, and coordinated baseline, midline, and endline quantitative surveys on nutrition and child development practices. Cornell University was responsible for ASTUTE’s operational research, which included TIPs (Trial of Improved Practices) studies designed to shed light on community acceptability of behaviours related to exclusive breastfeeding and complementary feeding [20,21].

In line with recommendations for social behaviour change communication (SBCC) and mass media campaigns, formative research was conducted to explore behavioural practices that contribute to stunting or factors that facilitate beneficial behaviours, and to develop the SBCC strategy. The results highlighted that many of the behaviours affecting outcomes of interest were associated with women’s social position and gender norms [22]. For example, a lack of male support during breastfeeding left women with inadequate time to breastfeed, leading women to give babies younger than 6 months additional fluids and complementary foods, which may contribute to stunting. The importance of engaging men in order to change behaviors associated with child stunting was confirmed in two further qualitative studies conducted in the target regions of Tanzania by ASTUTE partners [20,21]. Male involvement is increasingly recognized as a critical, yet underutilized, component of maternal and child health promotion. A growing body of evidence indicates that increasing men’s engagement with maternal and child health (MCH) can improve outcomes [23–26]. However, approaches to involve men can also have unintended negative effects. Formative research found that the Tanzanian policy of requiring women to attend their first antenatal care visit with their husband [27] hindered antenatal care (ANC) attendance and resulted in delayed initiation of ANC as women had to wait until their husbands agreed to attend [22,28]. Other barriers to positive behaviours associated with child stunting, in Tanzania and elsewhere, include cultural beliefs, stigmatization of men involved in MCH [29–32] and fear of taking HIV tests [28]. Men typically have less direct engagement with MCH services, making it difficult to reach them with important MCH and infant and young child feeding (IYCF) messages, indicating mass media as a potentially effective tool for reaching men.

In light of these findings, the ASTUTE mass media campaign was designed to create a shift in gender-related social norms relating to MCH. Therefore, a number of the ASTUTE SBCC messages were aimed directly at men (Table 1) with the intention of encouraging them to adopt behaviours supporting the health and well-being of their wives and children. The SBCC campaign used radio and television (TV) to target a range of practices affecting stunting, including promoting maternal health and nutrition during pregnancy, exclusive breastfeeding, complementary feeding, hygiene practices, and the treatment of diarrhoea. 60-second radio spots were broadcast 10 times a day on five regional radio stations and one national radio station; a new spot was aired each week. TV spots were broadcast on two national channels; they were aired twice a day, in the evening during the most-watched programs.

**Table 1:** SBCC campaign messages.

Themes and Sub-Messages	Barriers	Key messages to men
**1. Maternal Nutrition**		
1.1 Women planning to become pregnant and those who are currently pregnant should eat a nutritious diet.	Men control financial resources. Most men lack knowledge of maternal and child nutrition because they don’t attend ANC where maternal and child health education is provided.	Inadequate nutrition during pregnancy impacts not only the woman’s health but also the health of the unborn baby. Children of malnourished women are more likely to suffer from low birth weight, cognitive impairments, stunting, lower resistance to infections, and to have a higher risk of ill-health throughout their lives.
1.2 During pregnancy, women should get more rest.	Women are responsible for domestic and agricultural activities even during pregnancy. Women lack decision making power. Men consider women’s work as ‘exercise’ and believe that women’s work is beneficial, even during late stages of pregnancy.	Heavy physical activities increase the risk of complications during pregnancy and the possibility of delivering premature babies. Husbands should assist with household chores like fetching water and agricultural activities.
1.3 Women should attend ANC as soon as they realize they are pregnant and need to make sure they take the supplements and medicines provided.	Lack of male support to accompany women to ANC. Women who are not accompanied by their husbands at the first ANC visits are sent back home.	Husbands should accompany women to their first ANC appointment; Attending ANC early in pregnancy is important as it allows for early diagnosis and treatment of infections and conditions such as anemia. ANC provides access to immunization, vitamin supplements and preventative treatment for malaria which help to keep both the mother and unborn baby safe and healthy. ANC is important for monitoring the health of the mother and ensuring that the baby is growing well.
**2. Exclusive breastfeeding**		
2.1 Wait until the child is six months old before giving her anything but breastmilk. 2.2 Breastfeed more frequently (including emptying one breast before feeding from the other) to maintain sufficient supply.	The lack of support from men and other family members pushes women to resume household chores and agricultural work, soon after delivery (2–3 months). Mothers feel they have to introduce other foods so that they are free to work.	Men should assist with agricultural and household activities so that women have enough time to breastfeed. Breast milk protects babies from illness and helps them to develop. Giving babies anything other than breast milk in the first six months increases the risk of diarrhoea and other illnesses and hence increases the risk of death or disability.
**3. Complementary feeding**		
3.1 Feed children aged 6–23 months at least 3 nutritious meals per day with healthy snacks in between. 3.2 Starting at 6 months, continue to breastfeed your child, but add animal source foods such as eggs, poultry, and fish; green leafy vegetables; orange-fleshed foods such as vitamin A-rich fruits and orange tubers.	Men control the money available for purchasing food for the family and decide what produce (crops, small livestock, eggs) is owned and sold.	In order to grow well, children need a variety of nutritious meals throughout the day. Nutritious food makes children healthier, stronger and smarter.
**4. Early Childhood Development (ECD)**		
4.1 From birth, stimulate your child by telling him or her about objects at home and your interactions with others. Play, Talk, Praise.	Fathers think that it is the responsibility of mothers to play and talk to children. Fathers claim to not have time to spend at home with their children.	Early stimulation is important for the nourishment and growth of a baby’s brain. Children learn more quickly during their early years than at any other point in their lifetime.

While numerous empirical studies in Tanzania have used quantitative approaches to document maternal and child health (MCH) and nutrition outcomes, limited attention has been paid to the social and cultural determinants of these outcomes, particularly the role of fathers and male caregivers. Over the course of the three-year mass media campaign, we conducted a qualitative process evaluation using focus group discussions (FGDs) to assess the impact of the campaign on knowledge, attitudes, norms and behaviours. A separate quantitative evaluation [31] measured the impact of the campaign through a series of cross-sectional surveys. This paper focuses on the qualitative findings relating to the impact of the campaign on men’s knowledge, attitudes, norms, and behaviours. These findings highlight the importance of integrating males into strategies aiming at improving maternal and child health and nutrition outcomes.

## Methods

We conducted a series of fifty-nine (59) FGDs with target audiences across the five Lake Zone regions: Kigoma, Kagera, Geita, Mwanza, and Shinyanga. Each FGD consisted of 8–12 participants. FGDs included different participants in each iteration, and they started on 5^th^ October 2017, three months after the start of radio broadcasting, and continued until 1^st^ February 2020. We used the FGDs to explore people’s reactions to the campaign as well as possible mechanisms of behaviour change.

### Ethics statement

Research ethics clearance (NIMWHQ/R.8a/Vol.IX/2344) was obtained from the National Institute for Medical Research (NIMR) prior to the start of the campaign. Local protocols for entry into the villages were adhered to at the regional, district, ward, and village levels.

Participants were informed prior to the discussion that their involvement was voluntary. They received complete information about the research purposes and how their responses would be used and stored. All participants provided voluntary oral consent, which was recorded prior to the discussions. Their names were not recorded to ensure anonymity. The ethical guidelines for health research in Tanzania, as provided by the National Institute for Medical Research, permit the use of verbal consent when the study is deemed to be of minimal risk. Our study did not involve any medical procedures. Following the discussion, participants received a bar of soap as an appreciation for their time.

### Sampling and recruitment

Through previous formative research, and the application of Social Ecological Model (SEM) [33] we identified three key audiences for the campaign: (1) pregnant women and mothers of children aged under two years who serve as primary caregivers(Individual level); (2) husbands of pregnant women or fathers of children aged under two years, who are often heads of household and key decision makers(interpersonal/household level); and (3) elders, such as mothers-in-law, who often influence decision making and give advice to new mothers on parenting behaviours such as infant feeding (community level). Participants were recruited from each of these three audience segments. FGDs were conducted with homogeneous groups (e.g., women only) to encourage free expression of opinions.

We sought the assistance of District Nutrition Officers to identify villages for inclusion. Selected villages represented a range of contexts – a mixture of urban, peri-urban, and rural areas – and were not revisited. All villages were in areas covered by the radio broadcasts, and villages were selected from all five Lake Zone regions.

Within villages, purposive sampling was used to select participants for the FGDs. Community health workers in each village were informed in advance of the visit and were asked to identify and invite eligible individuals to participate. Mothers were eligible if they had children under two years (regardless of their marital status) or if they were pregnant. Men were eligible if they had children under two years and/or had partners/wives who were pregnant. Elders aged fifty and over were eligible, irrespective of household composition. To avoid bias during sampling, during each visit, which represented one district, researchers randomly selected three villages/ neighbourhoods. The selection of villages considered three characteristics: urban, peri-urban, and rural. Additionally, our sample size was substantial, with data collected from 35 villages across the 3 regions and discussions done with more than 500 participants.

Health workers who were part of the recruitment process were not present during the discussions, and participants were separated by gender. Additionally, participants were free to choose what information they wanted to share and what they preferred not to disclose. They also had the option to withdraw any time during the discussion without giving a reason.

### Data collection techniques and tools

SEM provides a framework to understand how individual behaviours are influenced by multiple, interacting levels of influence: individual, interpersonal, community, organizational, and societal [33]. This model is useful for examining maternal and child health behaviours, which are shaped by personal beliefs, household dynamics, social norms, and national policies. We drew on the SEM to guide our development of the interview guide used for Focus Group Discussions (FGDs) and to frame our interpretation of the campaign’s impact on male caregivers across different levels of influence.

The FGDs were led by two female researchers. A semi-structured question guide based on formative research, including a literature review, was used to facilitate the discussions **(****S1 Appendix****)** and was adapted as the study progressed. The guide included questions relating to radio listenership and television viewing habits, recall of radio and television spots, opinions on SBCC/mass media messages, and open-ended questions about actions taken as a result of hearing the spots. The discussions were conducted in places convenient for participants, either at a village hall, school, or health centre. Each FGD lasted approximately 45–60 minutes and was audio-recorded with participants’ consent; in addition, field notes were taken during the discussions. District Nutrition Officers attended the FGDs as observers and were available to answer questions relating to nutrition afterwards.

### Inclusivity in global research

This study ensured the engagement of the communities and that the communities were valued throughout the research process. Additional information regarding the ethical, cultural, and scientific considerations specific to inclusivity in global research is included in the Supporting Information **(****S1 Checklist****).**

### Analysis

Thematic analysis (TA) was used to analyze data [34]. Data analysis was completed manually. Some codes emerged inductively from the data, while other codes reflected a theoretical component from SEM that informed the data collection topic guide. All FGDs were conducted and recorded in Swahili. FGD recordings were transcribed in Swahili by the two researchers (DK and VA), which allowed for further familiarization with the data. The development of codes began during data collection. After every field trip in specific villages, researchers wrote field reports that were later used to identify initial codes during data analysis. DK worked with field reports, field notes, and a portion of transcripts to develop initial codes from the data inductively. Codes were modified and added during code development. From the sample of transcripts, field reports, and field notes, a codebook with a list of codes related to males’ involvement and a description of what each code entails was created. Before creating a final codebook, codes were shared with other researchers and overlapping and similar codes were removed/combined. A final codebook was used to code the remaining data set. DK read all the remaining transcripts and applied codes accordingly. The generated codes were then again reviewed by DK, re-reading the transcripts to check for accuracy and to make sure that codes accurately represented the collected data. After all the transcripts had been coded, related codes were grouped/combined to create broader categories/themes. Three categories that relate to male involvement emerged from the data: (1) Male engagement in maternal health (2) Male engagement in child nutrition (3) Male engagement in ECD-related activities.

## Results

Fifty-nine FGDs were conducted, spread across all 5 campaign regions, involving a total of 553 participants (Table 2). Twenty-two FGDs were conducted with men.

**Table 2:** Areas and number of participants in FGDs.

Region	District	Number of Participants		
		Fathers	Mothers	Elders
**Mwanza**	**Ilemela**	8	8	9
	**Misungwi**	12	12	8
	**Nyamagana**	8	8	
	**Sengerema**	9	10	10
	**Magu**	9	8	
	**Ukerewe**	8	12	7
	**Kwimba**	12	10	11
	**Kwimba**	10	11	9
**Geita**	**Geita DC**	10	9	
	**Geita TC**	9	10	10
	**Chato**	11	12	12
	**Chato**	10	11	
**Shinyanga**	**Kahama**	10	10	
	**Shinyanga MC**	7	7	8
	**Shinyanga DC**	7	11	
	**Msalala**			
**Kagera**	**Karagwe**	8	7	12
	**Karagwe**	12	12	
	**Ngara**	8	6	10
	**Bukoba MC**		10	10
**Kigoma**	**Kigoma DC**	8	11	
	**Kasulu**	10	10	12
	**Kasulu**	10	12	7
	**Kasulu**	7	10	
TOTAL		**203**	**227**	**135**

### Findings

The full code book can be found in the supporting documents (S1 Table).

### Male engagement in maternal health

#### ANC attendance.

Fathers reported that radio and TV spots increased their understanding of the importance of supporting women when pregnant and a change in their sense of responsibility around maternal health. For instance, they had started accompanying their wives to the clinic for ANC. Several fathers reported accompanying their wives to the clinic for antenatal care not because it was a requirement, but because they wanted to do so:

*I did not know that if a woman is pregnant, it is important to accompany her to the clinic. I knew this after hearing on the radio. When my wife became pregnant, I accompanied her for the first visit, and I was so praised by the health workers. I felt so good, and whenever she wanted to go, I would go with her until she gave birth. (Father, Ilemela)*

*In the past, if a woman was pregnant and told her husband to go to the clinic he would refuse, the woman would go alone and when she reached the clinic she would not be served, she would be told to go back home and bring her husband, but now men understand, for example, if my wife gets pregnant I will be happy if she tells me to go to the clinic I will not hesitate, there is nothing bad about going there……* (*Father, Ngara)*

Mothers also reported changes in their partners’ willingness to attend the clinic with them, allowing them to access ANC earlier, since they did not have to wait to convince their husbands to go. In the past, they had to persuade men to go even for the first clinic visit, but now men are willing to go. Women also reported that the campaign encouraged them to speak to their husbands, who were more receptive having heard the radio message:

*After hearing the spots on the radio, we have been motivated to speak to our husbands about going with us to the clinic when we are going for antenatal care. After hearing the spots, our husbands have been accepting this exercise. (Mother, Karagwe)*

*Nowadays, they* [men] *are the ones asking, when are we going to the clinic? (Mother, Chato)*

Once men were willing to attend the clinic, this willingness was reinforced by the information they got from the health service providers. Fathers reported learning more from service providers about reproductive and maternal health. Many fathers said that they trust information and are willing to act when they hear from the health workers themselves, rather than being told by their wives. The majority of the mothers indicated that they feel happy when their husbands attend the clinic with them because they know they will be given extra information.

*In the past, men did not accept taking HIV tests, saying that they were not the ones carrying the pregnancy. But now if we tell them, they accept, because they understand the importance, because of the advice they receive when attending ANC. (Mother, Karagwe)*

#### Maternal nutrition.

Men also reported becoming involved in maternal nutrition by reminding their wives to take their supplements and supporting them to eat nutritious food. One father who heard radio spots about the importance of medicines and supplements during pregnancy reported that he stressed to his wife that she should take them instead of throwing them away.

*After hearing the spots, I learnt about the importance of anti-malaria tablets for pregnant women. These medicines protect pregnant women against diseases. I insisted my wife take them because at the beginning she was hesitant, saying that they were smelling bad, but I told her to take them, she used them from the first medicine to the last, and she did not get sick from the beginning of the pregnancy to giving birth. (Father, Ngara)*

*I have been following the advice from that spot. When my wife was pregnant, I tried so much to make sure she ate various types of food, and I saw the results for my children, whom I got after I got this information my wife gave birth to a healthy child who was 5 kilograms. (Father, Chato)*

#### Reducing pregnant women’s workload.

Fathers reported that after exposure to the radio and TV spots, they realised the importance of helping pregnant women by reducing their workload so that they have more time to rest and remain in good health. Men were more motivated to help their wives stay healthy during pregnancy and to give birth to healthier babies, in order to reduce the amount spent on treatments when children or wives get sick. Men described helping their wives with farming, fetching water, collecting firewood, and household activities that are usually considered female tasks like cooking or taking care of the children. Some men said that they do not care what other men or community members would say when they see them doing what is considered women’s work. FGDs documented various stories of fathers taking on ‘female’ activities after hearing radio or television messages:

*What I learnt from the spots and took into action is helping my wife when she was pregnant. I used to help my wife, and she gave birth to a fat baby, and the baby is bright*.” *(Father, Ngara)*

*Personally, when my wife was pregnant, I was doing most of the activities, for example, cooking, fetching water, and looking for firewood, and I always took my children to the clinic while she was doing other activities at home. (Father, Kwimba)*

*From the TV spots, I learnt something about pregnancy, when a woman is pregnant, especially when she is close to giving birth, she should have enough time to rest, and we should help her with household chaos. (Father, Chato)*

*The spot I heard about helping pregnant mothers changed me because in the past, I could not carry a hoe while my wife was around, I thought it was her duty, but after hearing that spot I started to help. I can help her carry our baby when we are going out of the home. (Fathers, Kasulu)*

Men’s help with chores was also reported by a majority of women who admitted receiving more support from their husbands when pregnant than in previous pregnancies as a result of the information delivered in the radio and TV spots. Some participants said it is not hard to convince men who have heard this information on the radio or TV to help them, unlike those who have not heard these messages at all.

*When my stomach became big, he stopped me from doing such activities, he told me to rest and wait until I gave birth. I stopped doing heavy activities three months before giving birth... He got this information from the radio, and sometimes he even tells me after hearing these kinds of messages. (Mother, Chato)*

*When my pregnancy reached seven months, I could just stay at home, and he was helping me to fetch water and firewood*. *(Mothers, Kasulu)*

### Male engagement in child nutrition

#### Giving mothers more time to breastfeed.

After learning the importance of exclusive breastfeeding, men reported reducing women’s workload so they have time to breastfeed. Men reported that a reason for helping their wives in this way, was that their children would remain in good health, which means less will need to be spent on treatments for children.

*We are helping mothers because most mothers start giving their children porridge before six months so that the baby can sleep and set her free to work. Helping mothers with work enables them to get time to breastfeed* [exclusively] *for six months. The information we are getting from the radio is very helpful in changing people. (Father, Shinyanga)*

*I was hurt, after realizing that I was not doing the right thing, creating enough time for my wife to breastfeed… I started thinking I have made mistakes in the past, which I cannot reverse, so I decided from the day I heard the spot that I will insist my wife has enough time to breastfeed and stop giving babies food before six months. (Father, Misungwi)*

During the discussions, mothers confirmed getting help from their husbands so that they get enough time to breastfeed. Mothers expressed that the motivation from fathers is that they will have healthy babies.

*My husband tries to help me so that I can breastfeed. In the past, they would give us a lot of work, which meant we would breastfeed in a hurry, but now things have changed, they help us. (Mother, Bukoba)*

*I am given time to rest because even my husband wants a clever child; men get this information from clinics, but also from the radio. (Mother, Kasuru)*

#### Encourage exclusive breastfeeding.

After gaining knowledge on the importance of breastfeeding, fathers reported supporting, encouraging, and trying to convince women to breastfeed exclusively for six months. According to the participants, the media messages they heard helped to dispel the belief that breast milk may not be enough nutrition during the first six months. They also spoke of learning techniques to increase breast milk production, such as breastfeeding more frequently.

*I have a five-month-old child, I wanted to start feeding porridge, but from what I heard on the radio, I had to make sure the baby breastfeeds for six months without giving anything else. (Fathers, Ukerewe)*

*Two days ago, I went to visit my friend. We were betting, there was a baby there who had been given pineapple; she had pineapple in her mouth, then the father started complaining why are they giving her food, yet he had stopped them from giving food. While we were discussing a spot played on the radio, I asked them, Do you hear? A baby should not be given any food, even water, until she reaches six months. (Father, Geita)*

*I was so hurt because I did not know that children should have enough time to breastfeed. When I got that information, I intended to insist on my wife breastfeeding for six months without giving any food to the child. My wife believed that breastmilk alone is not enough and should be supplemented by porridge, but now we understand. (Father, Misungwi)*

*My first child was not able to breastfeed exclusively because my wife said she did not have enough breast milk. But when I learnt that breast milk can be produced the more a mother breastfeeds, I told my wife and we have done that with our second born and she is doing fine, and the breast milk is enough. (Father, Misungwi)*

#### Complementary feeding.

After gaining knowledge about nutritious food for children who are over six months, men reported making an effort to ensure their children get nutritious food, by saving money to buy a variety of nutritious foods.

*Fathers are the ones who manage economic resources. If I heard the importance of dagaa (small fish), for example, and I had planned to drink two beers, I would drink one beer and save some cash for the baby. Therefore, these spots remind us that if I happen to see an egg, I will say let me take one so that it will be mixed in the baby’s porridge. (Father, Ngara)*

*I started to follow all that I’m hearing from these spots on the radio about breastfeeding. I also heard on the radio that we can make her special food. I look for groundnuts to mix in her porridge, fish we mix with potatoes and mash together, and feed her… (Father, Kahama)*

Fathers reported that they passed on information they heard on the radio or TV to their wives, many of whom were busy with household chores and farming activities and lacked time to listen to the radio. A few fathers reported recording the spots on their phones and then sharing them with their wives.

*That day, my wife was not at home. I heard that spot, and when she came back from the farm, I told her I heard a spot. Luckily, I had recorded the spot in my phone, so when she arrived, she listened to the spot I recorded. (Father, Kasulu)*

### Male participation in ECD-related activities

#### Engaging with children.

Fathers reported engaging with their children as a result of information from the radio and TV spots. Fathers discussed their ways of showing love and engaging with their children, such as talking with them, playing with them, making toys for them, taking a short walk with their children, bringing them gifts when they go out, taking time to teach them how to count, reading, and showing pictures. They said they do this because every parent wants a smart and a healthy child.

*I learnt from one of these spots that I should start talking to a child when he is young. I like that spot because when you play and talk with a child, he/she will learn how to talk…even today, I was taking my child to the clinic.* (Father, Chato)

*As a father, I now understand that if I become harsh to my children, they will fear me……. I have learnt that I should be close to them and play with them. If you build an environment for children to fear you, even their brains will not develop well*. *(Father, Karagwe)*

#### Benefits of engaging with children.

Fathers also expressed the benefits of being close to their children, which include children becoming happy and free with their parents, children becoming close to their fathers and wanting to spend more time with them, children becoming smart/clever, shown by their knowledge of more things at a younger age, and children becoming happy and lively.

*When I started hearing the spots, I had a child who is eight months old now. I started teaching her when she was still very young. As she grows up, she does everything I teach her in practice, for example, if you see her now and tell her to raise her hands up, she will do so………. Playing with a child helps a child to learn. My child started calling her father when she was too young, it is because I played with her, talked to her, and taught her how to mention some things. (Fathers, Ilemela)*

*Parents have been benefiting from these spots because when you play with a child even when she is very young she will start recognizing things around her, for example I have a child who is seven months if you call her even when she was crying she will start laughing because I always play with her…if you hear those radio spots and take action you will have a bright baby. (Father, Karagwe)*

*I have a four-month-old baby, and the baby is happy and lively, and this is because of playing with my child. If we are keen on this, we will get educated people. (Father, Karagwe)*

*…I have a child who is three years old now, I decided to take a step to talk and play with him, playing with him makes him lively, I see the difference from my other children because this one is too clever. (Fathers, Ilemela)*

## Discussion

This qualitative study was conducted as part of a mixed-methods evaluation of a three-year mass media campaign targeting behaviors and practices affecting maternal and child health and nutrition. Findings indicate that the inclusion of radio and TV spots targeting men contributed to the success of the campaign across a number of domains. The campaign raised awareness among men about their involvement in the health of pregnant women and their children, making them more willing to accompany their wives to the clinic for ANC. This, in turn, had the benefit of exposing them directly to health information from health care workers. Men were also more willing to support their wives while pregnant and breastfeeding by helping with household chores, and providing nutritious foods for the mother, and for the baby when complementary feeding. Additionally, men played a role in disseminating the campaign information to their wives and other family members, thus increasing the reach of the campaign. Overall, the campaign appears to have helped change men’s perceptions that pregnancy and taking care of children are solely women’s affairs.

The themes emerging from our analysis reflect the multi-level nature of behaviour change as described in the Social Ecological Model [33]. At the individual level, fathers reported improved knowledge and personal motivation to support their partners. At the interpersonal level, changes occurred within the household, including communication between mothers and fathers, support for exclusive breastfeeding, and ANC attendance. At the community level, participants reported a shift in social norms, with men increasingly seen performing tasks considered women’s responsibilities. The organizational level was represented in interactions with ANC providers, where men received praise and further health education. This supports national policies that require male attendance at ANC and the campaign may have helped to normalise compliance with these expectations. Finally, at the societal level, mass media and government policy worked together to promote and normalize male involvement in maternal and child health.

Our findings indicate that the mass media campaign increased men’s willingness to attend ANC early with their wives and that this contributed to women’s increased ANC attendance. This is important because government policies [27] require men to attend ANC (especially the first ANC visit) with their wives, and studies in Tanzania and other African countries have reported delays in women initiating ANC due to male refusal to attend ANC with their wives [22,28]. These findings were confirmed in our quantitative evaluation of the campaign, which found that exposure to the campaign significantly increased women’s attendance for ANC by 10 percentage points, compared with those not exposed [31].

More generally, our findings support the benefits of men attending ANC with their wives and suggest some of the mechanisms through which men’s attendance may positively influence a range of maternal and child outcomes. In particular, men’s attendance resulted in direct exposure to health counselling by health workers, which had positive effects: men were more likely to perform health-related behavior when told by health workers and reluctant to act when they were told by their wives, as has been reported in another study in central Tanzania [35].

The campaign appears to have successfully mitigated some of the barriers arising from gender norms and women’s social position. For example, in response to the campaign, some fathers reported that they were now helping their wives with household activities so that wives had time to breastfeed or maintain their health during pregnancy. These findings were confirmed in the quantitative evaluation of the campaign, which found that men exposed to the campaign were significantly more likely to have helped with chores during their partner’s last pregnancy [31]. However, while the ASTUTE campaign directly targeted messages at men, encouraging them to assist their wives with household activities, the campaign’s impact on men’s behavior seems likely to have extended beyond the specific behaviors targeted in the campaign. Getting men more involved in maternal and child health may have contributed to broader shifts in gender roles, as has been found elsewhere [30]. However, despite the bulk of evidence from the FGDs and quantitative evaluation [31], indicating that the campaign had successfully mitigated some barriers arising from gender norms, it was also evident that gender and patriarchal norms continue to exert an influence on behaviours that negatively affect child nutrition, which may require long-term, multi-level interventions beyond media exposure.

The findings confirm that the campaign increased men’s knowledge about the importance of breastfeeding. Fathers reported encouraging women to breastfeed and applying pressure for exclusive breastfeeding for the first six months. The significance of breastfeeding for both the child and the mother has been highlighted, including increasing maternal attachment [36], delaying a new pregnancy, helping the uterus to return to its previous size, and protecting the baby against many infections [2].

Our findings indicate that the ASTUTE campaign successfully generated information diffusion within the target communities. Evidence from other campaigns [37–40] indicates that ‘social diffusion’ – information sharing and discussion within social networks- is an important part of the process through which social norms are shaped, and information is translated into practice [40]. It seems likely that the information diffusion generated by ASTUTE will have contributed to the underlying shift in gender-related social norms that appears to have occurred during the campaign. Researchers might consider capturing data on information diffusion when designing future campaign monitoring and evaluating activities.

### Strengths and limitations

Strengths of our study include its size (59 FGDs involving over 500 participants) and geographical spread (35 villages spread across five regions). Data on changes made as a result of the intervention were self-reported, and thus likely influenced by social desirability bias. The effects of social desirability bias is a potential weakness in all studies of this nature. However, all FGDs were facilitated by experienced researchers who took steps to mitigate social desirability bias, for example by asking about reasons why people did not carry out the targeted behaviors. Also, because FGDs were conducted separately with men and women, we were able to check for consistency between what was said by the male and female participants. Additionally, we have been able to use the findings of a large quantitative evaluation of the ASTUTE campaign to confirm some of the changes in behavior reported in our study.

We acknowledge the limitations of the study’s ability to assess the sustainability of the changes. However, since the completion of the ASTUTE project, DMI has continued to do child survival campaigns and feedback research in various villages, which confirms that the changes highlighted in this paper have been maintained over time. For example, fathers accompanying their wives to the clinic for ANC.

## Conclusion

Overall, our findings add to the evidence that interventions that target men’s behaviour can have beneficial effects on maternal and child health and add further insights into the ways that mass media campaigns can bring about behaviour change. In particular, radio and TV messages targeting men appear to be able to mitigate some barriers arising from gender norms and women’s social position. The ability of campaigns to generate ‘social diffusion’ of messages may well be important for campaigns aiming to shift social norms.

Existing evidence on the impact of interventions targeting maternal and child health predominantly relates to outcomes in women and children, even when the intervention incorporates components targeting men or couples. Given the importance of changes in men’s behaviour, increased reporting on male behaviour changes in future evaluation studies would be beneficial. Creating gender-sensitive mass media campaigns that specifically target men can help challenge harmful gender norms and encourage men’s participation in caregiving, which would subsequently increase the utilization of maternal and child health services.

## Supporting information

S1 Appendix Semi-structured interview guide. (DOC)

S1 Checklist Inclusivity in global research. (DOCX)

S1 Table Final code book. (DOCX)
