# Supplementary material for: Targeting men to improve maternal and child health and nutrition: A qualitative process evaluation of a mass media campaign in Tanzania’s Lake Zone
Source: PLoS One. 2026 Jan 2;21(1):e0338437. doi: 10.1371/journal.pone.0338437 (PMC12758809; doi:10.1371/journal.pone.0338437)
Supplement: S1 Appendix — (DOC) [file pone.0338437.s001.doc]

It is expected that you will discuss the radio spots with at least 3 different groups (dependent on time and budget available) 1) a group of women (with children under 2 years & pregnant women), 2) a group of men (with children under 2 years & pregnant wives), and 3) a group of elders. Groups 1 and 2 can be a mixtures of married, unmarried, adolescents, and people with children. Each group should consist of 8-10 people. The following information should be recorded for each focus group session:

**Time and date:**

**Location:**

**Brief description of constitution of focus group:**

**Introduction**

Hello good morning/afternoon. My name is ............ and I work with ASTUTE, an organization that creates television and radio spots. We produced some new radio/TV spots on the topic of nutrition and we would like to find out if you’ve heard these spots and if so, what you thought about them.

Introduction of participants: Before you start, browse the group and invite each participant to introduce themselves giving their names, jobs, and the number of children.

Our discussion will last around 30-45 minutes and what you tell us will remain confidential. We will not record your names. We would like to hear your opinions about the radio spots (or television) we've produced so we can be sure that these spots are suitable for an audience like yourselves.

We'd love to hear your thoughts, beliefs and experiences relating to these spots. We are not here to teach or educate you. There are no right or wrong answers, and you are encouraged to say what you want. You can express positive and negative ideas, you can say you do not agree with the expressed opinions of others, and you can always change your mind.

It is very important for us to hear what each of you think, so try to give everyone a chance to speak freely. Please, avoid small conversations with your neighbour, so that everyone can hear what is said.

From our side, we will not give you our opinion. Our role is to guide the discussion so that everyone has a chance to participate and speak, and verify that all discussion topics are addressed. We have lots to discuss let’s get on.

Do you have questions? (Obtain signed consent at this point)

***Listening to DMI radio/TV broadcasts***

1. Do you listen to the radio or television (insert name of our partner radio/TV stations in the region)? (Keep a count of the number of listeners/viewers for each station)

2. Have you ever heard radio/TV spots on nutrition on any of these stations? (Keep a count of the number of listeners for each station)

**Nutrition sub topics:**

- Maternal nutrition
- Exclusive breastfeeding
- Complementary feeding
- WASH
- Early childhood development

3. Describe the messages/stories you have heard/seen in these spots. What were the health issues raised/addressed? Get participants to recall specific spots wherever possible (We need to check the plot and content of the spots, so we can be sure they are definitely referring to DMI spots.)

**Note:** If people begin to describe other nutrition radio spots, it is still useful to record details of the spots they mention, so that we have information on what the other spots are broadcast. But you must be clear when continuing on to the detailed questions that respondents are referring only to the ASTUTE spots.

***Repeat questions below separately for each specific (ASTUTE spots) radio /TV spot described by participants***

4. On what station(s) radio have you heard this spot?

5. At what time of day did you hear this spot? And how often?

6. In your opinion, who do you think this spot is targeted towards? (Explain)

7. What did you like about this spot? (Explain)

8. What did you not like about this spot? (Explain)

9. Do you think that people (who hear this spot) will follow the advice/message given in the spot?

- If so, who and why especially? - If not, who mainly and why?

***After discussing each of the specific spots recalled by members of the group, the following more general questions should be posed to the whole group.***

10. In your opinion, since the ASTUTE nutrition messages have been broadcasted, do you think people have changed their behavior? If so, explain who and why. If not, also explain why.

- In your opinion, what do you think are the main obstacles that continue to prevent people in this community from changing?

-What do you think would encourage or motivate more people to adopt these behaviours?

11. Since you listened to the messages in this spot, have you changed your behavior accordingly (Record examples explaining how they have changed and why/why not? Explore causality related to the spots, obtain testimonials).

***Finally, please thank the participants for their time!***
