# Supplementary material for: Targeting men to improve maternal and child health and nutrition: A qualitative process evaluation of a mass media campaign in Tanzania’s Lake Zone
Source: PLoS One. 2026 Jan 2;21(1):e0338437. doi: 10.1371/journal.pone.0338437 (PMC12758809; doi:10.1371/journal.pone.0338437)
Supplement: S1 Table — (DOCX) [file pone.0338437.s003.docx]

**LIST OF CODES AND THEIR DEFINITIONS**

| **Category** | **Code** | **Description** | **Example from data** |
| --- | --- | --- | --- |
| Man’s engagement in maternal health | Attending ANC | This captures all information related to the involvement of men in their partner's attendance of ANC | In the past, if a woman is pregnant and tells his husband to go to the clinic he will refuse, the woman will go alone and when she reaches the clinic she will not be served she will be told to go back home and bring his husband, but now men understand, for example now if my wife gets pregnant I will be happy if she tells me to go to the clinic I will not hesitate, there is nothing bad about going there…… [Father, Ngara]  “I did not know when a woman is pregnant I should go with her to the clinic, I knew this after hearing from the radio, when my wife was pregnant I accompanied her the first visit, and every time she needed my company I would go with her” [Father, Ukerewe]  I think fathers have now changed in the past, if a woman is pregnant and tells his husband to go to the clinic he will refuse, the woman will go alone and when she reaches to the clinic she will not be served she will be told to go back home and bring his husband, but now men understand, for example now if my wife gets pregnant I will be happy if she tells me to go to the clinic I will not hesitate, there is nothing bad about going there*……*  [Fathers, Ngara)  “After hearing the spots from the radio we have been motivated to speak to our husbands about going with us to the clinic when we are going for antenatal care, after hearing the spots our husband have been accepting this exercise” (Mothers, Karagwe))  “In the past a man would not allow to go to the dispensary for HIV testing, he would say he is not pregnant, but now if you tell him to go he goes” [Mothers, Karagwe]  “Yes they have received this information from the media but also after they agreed to start attending ANC it means that they also receive advice from health service providers, and they full fill what they have been instructed.” (Mother, Karagwe)  “In the past men did not accept taking HIV tests saying that they are not the ones carrying pregnancy, but now if we tell them they accept because they understand the importance, because of the advice they receive when attending ANC.” (Mother, Karagwe) |
|  | Maternal nutrition | This captures information related to the males involvement in making sure that pregnant women receive all important nutrients | “I have been following the advice from that spot, when my wife was pregnant I tried so much to make sure she eats various types of food, and I saw the results for my two children whom I got after I got this information, my wife gave birth to a health child who was 5 kilograms.” [Father, Chato]  “After hearing the spots, I learnt about the importance of anti-malaria tablets for pregnant women. These medicines protect pregnant women against diseases, I insisted my wife take them because at the beginning she was hesitant, saying that they are smelling bad, but I told her to take them, she used them from the first medicine to the last and she did not get sick from the beginning of the pregnancy to giving birth.” [Father, Ngara] |
|  | Reducing pregnant women’s workload | Captures all information related to the involvement of men to ensure that their partners get time to rest during pregnancy | “They teach how men should treat pregnant women, helping them with household activities, if a woman is breastfeeding should be given more time to breastfeed.” [Fathers­, Kasulu]  “What I learnt from the spots and took it into action is helping my wife when she was pregnant. I used to help my wife and she gave birth to a fat bay and the baby is bright.” [Fathers, Ngara]  “Personally, when my wife was pregnant, I was doing most of the activities for example cooking, fetching water, looking for firewood and I always take my children to the clinic while she is doing other activities at home.” [Father, Kwimba]  “From the TV spots I learnt something about pregnancy, when a woman is pregnant especially when she is close to giving birth should have enough time to rest, we should help her with household chaos.” [Father, Chato]  “When my stomach became big he stopped me from doing such activities, he told me to rest and wait until I give birth, I stopped doing heavy activities three months before giving birth….he got this information from the radio sometimes he even tells me after hearing these kinds of message.” [Mother, Chato]  “When I was six months pregnant he told me I should not do heavy activities, I should have more time of resting; he said if I do heavy work I might get problems.” [Mother, Chato]  “The spot I heard about helping pregnant mothers changed me because at in the past I could not carry a hoe while my wife is around, I thought it was her duty but after hearing that spots I started to help, I can help her carry our baby when we are going out of home” [Fathers, Kasulu]  “When my pregnancy reached seven months, I could just stay at home he was helping me to fetch water and firewood” [Mother, Kasulu]  “What I learnt from all these spots is that all activities which previously I considered as women’s work, personally I don’t believe in that anymore. I don’t feel shame washing clothes, taking care of the children, cleaning a child when he/she needs to be cleaned, and as a result of that, my children cannot differentiate between mother’s love and father’s love.” [Father, Karagwe]  “My sister in-law who was pregnant was doing very heavy activities, she used to carry a bucket of water on her head and another one on her hand, I advised her to be fetching water early in the morning and then have a rest and she followed the advice, I gave his husband my own bicycle and told him to use it for fetching water in the morning before he leaves for work.” [Father, Shinyanga]  “I gave advice to my young brother whose wife was pregnant when it was cultivation season, he used to go with his pregnant wife to the farm I stopped him and advised him to look for another person who will be helping with all the farming activities, my young brother agreed and the wife gave birth without any problem.” [Father, Shinyanga] |
| Men’s engagement in child nutrition | Giving women more time to breastfeed | This captures information related to all information related to the engagement of men in creating time for mothers to breastfeed | “My husband tries to help me so that I can breastfeed. In the past they would give us a lot of work, this meant we would breastfeed in a hurry, but now things have changed, they help us…” (Mother, Bukoba)  “If a woman is breastfeeding should be given more time to breastfeed.” [Fathers, Kasulu]  “I am given time to rest because even my husband wants a clever child, men get this information from clinics but also from the radio” (Mother, Geita)  *“*After listening to the spots, I have reduced workload from my wife, some of these were considered to be women’s duties. I don’t see anything bad if I wash clothes or to clean our 10 months child now…” *(Father, Nyamagana)*  “What made me start helping my wife is because of the information I heard from the radio, children I had before I had this information were not in good health, but when I started to help I also see the health of my children is getting better it is because in the past I did not have this information.” **[**Fathers, Shinyanga]  “We are helping mothers because most mothers start giving their children porridge before six months so that the baby can sleep and set her free to work. Helping mothers with work enables them to get time to breastfeed [exclusively] for six months. The information we are getting from the radio is very helpful in changing people.” [Father, Shinyanga]  “Personally, I was hurt, after realizing that I was not doing the right thing, creating enough time for my wife to breastfeed… I started thinking I have made mistakes in the past which I cannot reverse so I decided from the day I heard the spot that I will insist my wife has enough time to breastfeed and stop giving babies food before six months.” [Father, Misungwi] |
|  | Encourage exclusive breastfeeding/ | This captures information related to the efforts that men make to make sure that women breastfeed exclusively for six months | “…I have a five months old child, I wanted to start feeding porridge but I heard on the radio, I had to make sure the baby breastfeeds for six months without giving anything else…” (Fathers, Ukerewe)  “Two days back I went to visit my friend we were betting, there was a baby there who had been given pineapple, she had pineapple in her mouth, then the father started complaining why are they giving her food yet he had stopped them from giving food, while we were discussing a spot was played in the radio, I asked them do you hear? a baby should not be given any food even water until she reaches six months.” [Father, Geita TC]  “There was one parent who came to my house with a baby, the baby was crying a lot and I saw her giving the baby water, I stopped and asked her why are you giving this child water yet she is not even six months, haven’t you heard in the radio they say if the child is below six months should not be given any kind of food, should only be given breast milk, she breastfed the baby and the baby stopped crying.” [Father, Gita DC]  “I was so hurt because I did not know children should have enough time to breastfeed. When I got that information I intended to insist on my wife to breastfeed for six months without giving any food to the child, my wife believed that breastmilk alone is not enough and should be supplemented by porridge, but now we understand’.”  [Father, Ngara]  “When I heard the spot, I was touched because my child was already 8 months old. But my second child will exclusively breastfeed frequently so that he or she can develop fully and be protected from illnesses.” (Father, Misungwi).  “My first child was not able to breastfeed exclusively because my wife said she did not have enough breast milk. But when I leant that breast milk can be produced the more a mother breastfeeds, I told my wife and we have done that with our second born and she is doing fine and the breast milk is enough. (Father, Misungwi). |
|  | Complementary feeding | This captures information related to efforts that men make in making sure that their children get appropriate food after six months | “Fathers are the ones who manage economic resources, if I heard the importance of dagaa for example and I had planned to drink two beers I will drink one beer and save some cash for the baby, therefore these spots remind us, if I happen to see an egg I will say let me take one so that it will be mixed in the baby’s porridge.” [Father,_Ngara]  “I started to follow all that I’m hearing from these spots in the radio from breastfeeding. I also heard in the radio that we can make her special food, I look for groundnuts to mix in her porridge, fish we mix with potatoes and mash together and feed her…” (Father, Kahama] |
| Male engagement in ECD-related activities | Engaging with children | This captures information related to the men’s expressions about the activities they do to engage with their children | “When I was playing with my child football, someone came and told my wife that I am behaving like a kid, I told my wife let her talk, she did not know what she was talking about…….I am still playing with my child, the child is health and very charming.” [Fathers, Ngara]  “…I am raising my child well, I play with her and I am close to her. She is four years old and I see the fruits of playing with her, and I know it is because I heard the spots on the radio…” (Father, Chato)  “…I have a four month baby, the baby is happy and lively, and this is because of playing with my child. If we are keen on this, we will get educated people…” (Father, Karagwe)  “…I have a child who is three years now, I decided to take a step to talk and play with him, playing with him makes him lively, I see the difference from my other children because this one is too clever…” [Father, Ilemela]  “When I started hearing the spots I had a child who is eight months now, I started teaching her when she was still very young, as she grows up, she does everything I teach her in practice, for example if you see her now and tell her to rise her hands she will [Father, Ilemela]  “As a father I now understand that if I become harsh to my children they will be fearing me…….. I have learnt that I should be close to them, and play with them, if you build an environment for children to fear you even their brains will not develop well.” [Father, Karagwe]  “I learnt from one of these spots that I should start talking to a child when he is young, I like that spot because when you play and talk with a child he/she will learn how to talk…even today I was taking my child to the clinic” (Father, Chato)  “When my child cries, I don’t have to call her mother……when I want go out of home my children would want to go with me, I never stop them I will carry them go out with them for some minutes just to make him calm then call someone to carry him.” (Father, Chato) |
|  | Benefits of engaging with children | This captures information related to the fathers thought about all the benefits of fathers engaging with their children | “…I have a year and 6 months old child, my child loves me and is very lively and different from others. We are thankful for the spots and the CHWs’ work…” [Fathers, Karagwe]  “I am still playing with my child, the child is health and very charming.” [Fathers, Ngara]  “…I have a four months baby, the baby is happy and lively, and this is because of playing with my child. If we are keen on this, we will get educated people…” [Father, Karagwe]  “…mine is seven months, through playing with my child, I have realised that the child knows some of the things around the household…” [Father, Karagwe]  “…mine is one year and ten months now, she has become too clever and lively, I personally wonder where all that comes from.” [Father, Kahama]  “Playing with a child helps a child to learn my child started calling father when she was too young, it is because I played with her, talk and teaching her how to mention some things.” [Father, Ilemela] |
